# Toll-like receptor 4-mediated necroptosis in the development of necrotizing enterocolitis

**DOI:** 10.1038/s41390-021-01457-y

**Published:** 2021-03-17

**Authors:** Tianjing Liu, Haifeng Zong, Xiaoyu Chen, Sihang Li, Ziyun Liu, Xuewei Cui, Guoqiang Jia, Yongyan Shi

**Affiliations:** 1 Department of Pediatric Orthopedics, Shengjing Hospital of China Medical University, Shenyang, China; 2 Department of Neonatal Intensive Care Unit, Affiliated Shenzhen Maternity and Child Healthcare Hospital, Southern Medical University, Shenzhen, China; 3 Department of Pediatrics, Shengjing Hospital of China Medical University, Shenyang, China

## Abstract

**Background:**

Dramatic intestinal epithelial cell death leading to barrier dysfunction is one of the mechanism of neonatal necrotizing enterocolitis (NEC), in which Toll-like receptor 4 (TLR4) plays a pivotal role. This study explored the role of necroptosis, a drastic way of cell death in NEC.

**Methods:**

The expression of necroptotic proteins was tested in NEC intestinal tissue and compared with controls. NEC was induced in neonatal wild-type mice and a necroptosis inhibitor was given to investigate whether NEC could be relieved. The general condition, macroscopic scoring, and histological evaluations were performed. The expression of tight junction proteins, inflammatory cytokines, and necroptosis-related proteins was measured, and barrier function was examined. Then, NEC was induced in TLR4-knockout pups to confirm the role of TLR4 in necroptosis.

**Results:**

Necroptotic proteins were significantly upregulated in both NEC patient and animal models, together with the expression of TLR4. NEC could be relieved and inflammatory infiltration was decreased by necrostatin-1s. TLR4-knockout mice showed milder tissue degradation and less necroptosis after NEC induction.

**Conclusions:**

Necroptosis is an essential pathological process of NEC. TLR4 may be one stimulator of necroptosis in NEC. Inhibiting the intestinal cell necroptosis might be a useful strategy in the treatment of NEC.

**Impact:**

- Necroptosis is a key pathological process in NEC, which appears to involve TLR4.
- Anti-necroptosis treatment is a promising strategy that could significantly relieve the symptoms of NEC.

## Introduction

Neonatal necrotizing enterocolitis (NEC) is a concern due to the increased survival of preterm births. When the immature gut is not yet ready for the external environment, NEC may develop. The manifestations include feeding intolerance, abdominal distention, intestinal perforation, and long-term dysfunction of the involved section.^1^ Pathologically, there is epithelial sloughing, submucosal edema, neutrophil infiltration, and destruction of the villus architecture. NEC occurs in 7% of preterm infants with birth weights between 500 and 1500 g and kills 20–30% of the patients. A quarter of the survivors suffer from long-term complications such as short gut syndrome and neurodevelopmental impairment.^2^

Many factors predispose preterm neonates to NEC, including intestinal immaturity, enteral feeding, intestinal dysbiosis, ischemic injury, and inflammation.^3^ These factors lead to excessive intestinal cell death, subsequent barrier dysfunction, contact of the microbiome with the submucosal layer, and subsequent inflammatory responses. Apoptosis has always been regarded as a major mechanism of cell death in NEC and is a programmed, sequential, and immunologically silent process that has been demonstrated to cause barrier dysfunction in bowel diseases, including NEC.^4,5^ However, apoptosis is not completely suited to the explosive, devastating process associated with NEC, which prompts us to look for other mechanisms in NEC pathology. Notably, the pathological process of NEC is highly indicative of necroptosis, which has recently been distinguished from necrosis. Necroptosis involves the formation of pores in the cell membrane and the release of intercellular content, resulting in severe and drastic inflammation that strongly resembles the pathological changes in NEC.^6^

Toll-like receptor 4 (TLR4) has been demonstrated to be indispensable in NEC. Normally, the expression of TLR4 in epithelial cells gradually decreases after birth, but in murine models of NEC, TLR4 remains highly expressed.^7^ This high level of TLR4 increases apoptosis and autophagy and reduces proliferation and migration in both enterocytes and intestinal stem cells.^8^ Moreover, TLR4 is an activator of necroptosis. TLR4 can bind with TRIF (Toll/interleukin-1 (IL-1) receptor-domain-containing adaptor inducing interferon-β (IFN-β)) and then with receptor-interacting protein kinase 3 (RIPK3) to induce necroptosis.^9^ This finding suggests that the high level of TLR4 in NEC activates intestinal epithelial cell necroptosis, contributing to barrier dysfunction and local inflammation.

This study investigated the role of necroptosis in NEC patients and model animals. An anti-necroptotic agent was administered to NEC model animals to determine whether it could substantially alleviate NEC. The role of TLR4 as an initiator of necroptosis in NEC was also examined in a TLR4-knockout model.

## Materials and methods

### Human samples

This study was approved by the Ethnic Board of Shengjing Hospital of China Medical University (2016PS236K) and strictly followed the WMA Declaration of Helsinki. Caretakers of all participants signed written informed consent before participation. Intestinal tissue was collected from NEC patients who underwent enterectomy because of irreversible necrotic changes in intestinal segments. The tissue was collected randomly from the enterectomized bowel. The control group consisted of those who underwent enterectomy due to congenital intestinal atresia. In this group, the tissue was harvested from the end of the enterectomized bowel away from any ischemic lesions. The demographic data of the participants are listed in Supplementary Table 1.

### Animal preparation and NEC induction

All animal procedures were reviewed and approved by the Laboratory Animal Ethics Committee of China Medical University. This study was carried out in strict compliance with the approved protocols. TLR4-knockout mice with a C57BL/6J background were purchased from Jackson Laboratory (stock number 029015). Pregnant C57BL/6J mice were supplied by the Animal Lab of the Experimental Research Center of Shengjing Hospital, China Medical University. All animals were kept in specific pathogen-free static cages with a 12-h light/dark cycle. Chow pellets and tap water were available ad libitum. The sex ratio was 1:1 in each group.

NEC was induced by the formula feeding and cold/asphyxia stress method.^5,10^ Mouse pups weighing 3–4 g were separated from their mother beginning on the seventh postnatal day. The pups were kept warm and humidified in a newborn incubator. Neonatal animals were fed formula (Esbilac, 200 cal/kg/day; PetAg, Hampshire, IL) at a dose of 40 ml/kg every 4 h for 72 h. Moreover, the neonatal animals were challenged with hypoxia (5% oxygen and 95% nitrogen) for 3 min, followed by exposure to cold (4 °C) for 10 min twice a day for 3 days. Neonatal pups in the control group were breastfed by their mothers and kept at room temperature with normal air.

### Cell culture and treatments

The human intestinal epithelial cell line HCT-116 was cultured in Dulbecco’s modified Eagle’s medium supplemented with 10% fetal bovine serum at 37 °C and 5% CO_2_. Cells were transfected with human TRIF-specific short interfering RNA (siRNA) and/or human tumor necrotic factor receptor 1 (TNFR1)-specific siRNA using Lipofectamine 3000 (Invitrogen) to inhibit the production of these proteins. After the silencing effect had been confirmed by Western blotting, lipopolysaccharide (LPS) from *Escherichia coli* was administered at a concentration of 100 ng/ml to induce necroptosis.

### Necrostatin-1 treatment

Necrostatin-1s (nec-1s; Cat. #2263, Biovision, Milpitas, CA) was dissolved in 10% dimethyl sulfoxide (DMSO) and administered to the mice at a dose of 2 mg/kg by intraperitoneal injection once each day after the initiation of NEC. The control group was administered the same volume of 10% DMSO.

### Western blotting

For in vivo experiments, total protein was extracted from the distal ileum. For cell experiments, total protein was harvested with RIPA buffer according to the instructions. Equal amounts of proteins (50 μg per lane) were separated by 10% polyacrylamide gel electrophoresis, and the proteins were transferred electrophoretically onto polyvinylidene difluoride membranes (EMD Millipore, Billerica, MA). The membranes were incubated with the following primary antibodies: anti-TLR4 (ab13556, Abcam, Cambridge, MA), anti-pRIPK1 (mouse) (31122, Cell Signaling), anti-pRIPK1 (human) (65746, Cell Signaling), anti-pRIPK3 (mouse) (91702, Cell Signaling), anti-pRIPK3 (human) (93654, Cell Signaling), anti-pMLKL (mouse) (37333, Cell Signaling), anti-pMLKL (human) (91689, Cell Signaling), anti-caspase-8 (8592, Cell Signaling), anti-caspase-3 (9662, Cell Signaling), anti-zonula occludens-1 (ZO-1, 339100, Invitrogen; Thermo Fisher Scientific, Waltham, MA), anti-occludin (33-1500, Invitrogen), anti-claudin-1 (71-7800, Invitrogen), anti-claudin-2 (32-5600, Invitrogen), anti-TRIF (4596, Cell Signaling), anti-TNFR1 (3736, Cell Signaling), and anti-β-actin (A1978, Sigma-Aldrich, St. Louis, MO). Relative protein levels were quantified using ImageJ software (Version 1.52a, National Institutes of Health, Bethesda, MD).

### Histology

The distal ileum or proximal colon was collected and sectioned at a thickness of 4 μm. The slides were stained with hematoxylin and eosin (Beyotime Institute of Biotechnology, Haimen, China) according to the manufacturer’s instructions. Microscopic scoring was performed according to Nadler et al.^11^ with the following scale: score 0, intact intestinal structure and villi; score 1, separation of the villus core without other abnormalities; score 2, villus core separation, submucosal edema, and epithelial sloughing; score 3, exacerbated villus core separation, submucosal edema, and local denudation of epithelium with loss of villi; and score 4, extensive denudation of epithelium, complete loss of villi, full-thickness necrosis, or perforation. The average score of five fields was recorded as the final score of the pup. Two pathologists who were blinded to the study design performed the evaluations separately.

### Immunohistology and immunofluorescence

Immunohistology was used to investigate the local expression of pRIPK3 (1:200 dilution; 93654, human pRIK3, Cell Signaling). The slides were subsequently incubated with horseradish peroxidase-conjugated anti-IgG secondary antibodies. The stained slides were stained with 3,3′-diaminobenzidine substrate (Sigma-Aldrich) and observed under a light microscope.

Immunofluorescence was used to investigate the local expression of pRIPK3 (1:200 dilution; 91702, mouse pRIK3, Cell Signaling), CD4 (1:200 dilution; Santa Cruz Biotechnology Inc., Dallas, TX), and cleaved caspase-3 (1:200 dilution; 9661, Cell Signaling). The slides were subsequently incubated with Alexa Fluor 488 or 555 secondary antibodies (Invitrogen). The stained slides were visualized using a Leica DFC425 fluorescence microscope [Leica Microsystems (Schweiz) AG, Heerbrugg, Switzerland].

### Real-time PCR

Total RNA was isolated from the distal ileum using TRIzol reagent (Invitrogen). First-strand complementary DNA was synthesized with a PrimeScript RT Reagent Kit (Invitrogen). Real-time PCR was performed in a 20-μl reaction volume using a SYBR-Green PCR Reagent Kit (Takara Biotechnology Co., Japan) and a Bio-Rad IQ5 real-time system. The 2-ΔΔCq method was used to quantify the relative messenger RNA (mRNA) expression. β-2 microglobulin was used as an internal control. The sequences of the PCR primers are listed in Supplementary Table 2.

### Intestinal permeability measurement

This measurement was performed 48 h after NEC induction. The pups were fasted for 3 h before gavage. Fluorescein isothiocyanate (FITC)-conjugated 4 kDa dextran (FD4, 50 mg/ml, Sigma) was administered via gavage at a dose of 4 μl/g. Blood serum was collected 1 h later. Fifty microliters of sample per well was added to a 96-well plate, and the serum concentration of FD4 was measured using a Synergy HT plate reader (BioTek Laboratories Inc., WA).

### TUNEL staining

Sections of the distal ileum were examined. Terminal deoxynucleotidyl transferase-mediated dUTP nick-end labeling (TUNEL) staining was performed using an In Situ Cell Death Detection Kit with TMR red (Roche Diagnostics, Indianapolis, IN) according to the manufacturer’s instructions.

### Statistical analysis

The data are presented as the mean ± standard deviation (SD). The Mann–Whitney *U* test was used for comparisons between two groups with unequal variances. For comparisons of more than two groups, one-way analysis of variance or the Kruskal–Wallis test was performed. Dunnett’s *t* test was used to examine differences between two selected groups to adjust for bias caused by repeated comparisons. All statistical comparisons were performed using GraphPad Prism software version 6.0 (GraphPad Software 6.0, La Jolla, CA) and SPSS 23.0 (SPSS, Chicago, IL).

## Results

### NEC patients and animals had increased intestinal necroptosis and elevated TLR4 expression

**Fig. 1:**
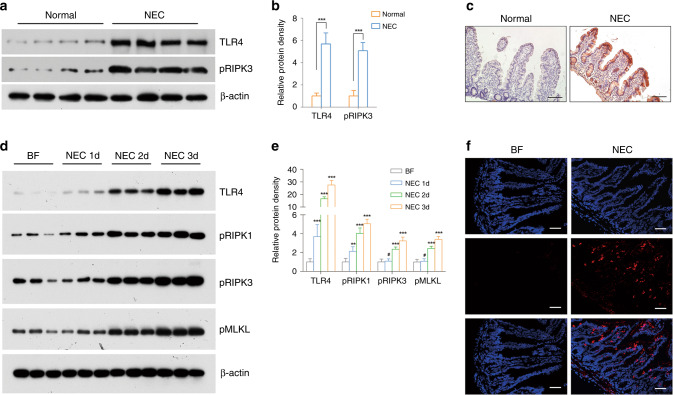
Necroptosis and TLR4 were upregulated in NEC patients as well as in model animals. **a** Western blotting and **b** relative protein density of phosphorylated RIPK3 and TLR4 in control and human NEC biopsies. *n* = 4 per group. ***p < 0.001. **c** Typical immunohistological staining of phosphorylated RIPK3 in controls and human NEC biopsies. Magnification = ×100, bar = 100 μm. **d** Western blotting and **e** relative protein density of phosphorylated RIPK1, phosphorylated RIPK3, phosphorylated MLKL, and TLR4 in control and NEC murine pups at a time sequence. *n* = 6 per group. **f** Typical immunofluorescent staining of phosphorylated RIPK3 in control and NEC animals. Magnification = ×200, bar = 50 μm. BF breastfeeding under a physiological status, NEC neonatal mice that underwent formula feeding and cold/asphyxia stress. ^#^*p* > 0.05, **p < 0.01, ***p < 0.001 comparing with the BF group.

Increased intestinal necroptosis was observed in human NEC bowel tissue, as demonstrated by increased pRIPK3 expression (*p* < 0.001) (Fig. 1a, b). Immunohistological analysis showed that this increase mainly occurred in the epithelium (Fig. 1c). Moreover, the elevation in TLR4 was observed in patients (*p* < 0.001) (Fig. 1a, b). In NEC mice, the expression of necroptotic proteins increased with disease progression, indicating the gradual activation of necroptosis in NEC. TLR4 also showed a similar trend, which confirmed its role in the progression of necroptosis of NEC (Fig. 1d, e). Immunofluorescence staining also showed a similar increase in pRIPK3 in the intestinal epithelium in NEC mice (Fig. 1f).

### The RIPK1 antagonist nec-1s ameliorated NEC in neonatal mice

**Fig. 2:**
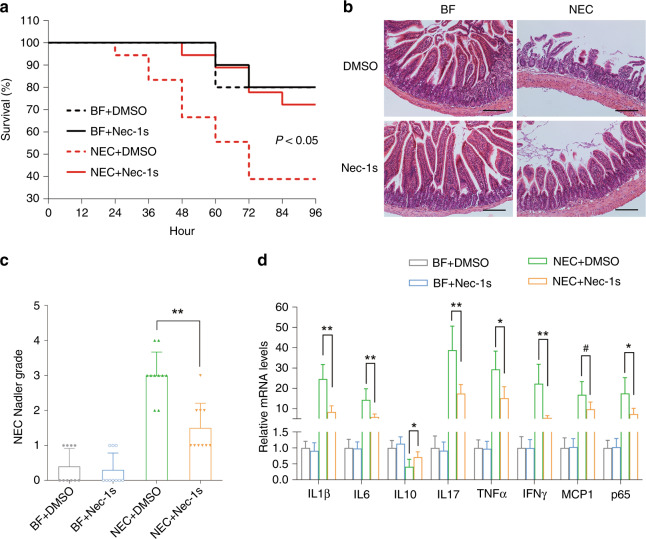
Necrostatin-1s ameliorated NEC in animal models. **a** The survival rate of neonatal mice in each group. *n* = 10 in either of the BF groups and *n* = 18 in either of the NEC groups. **b** Typical hematoxylin and eosin staining of the distal ileum in each group, magnification = ×100, bar = 100 μm. **c** The Nadler’s grade, *n* = 10 in each group. NEC + DMSO vs. NEC + Nec-1s, *p* = 0.001. **d** Real-time PCR showing the expressions of the inflammatory cytokines, *n* = 6 in each group. Statistical comparisons were done between the NEC + DMSO group and the NEC + nec-1s group. BF breastfeeding under a physiological status, NEC neonatal mice that underwent formula feeding and cold/asphyxia stress, DMSO 10% DMSO in PBS, the dissolvent of necrostatin-1s, nec-1s necrostatin-1s. NEC + DMSO vs. NEC + Nec-1s, ^#^*p* > 0.05, *p < 0.05, and **p < 0.01.

**Fig. 3:**
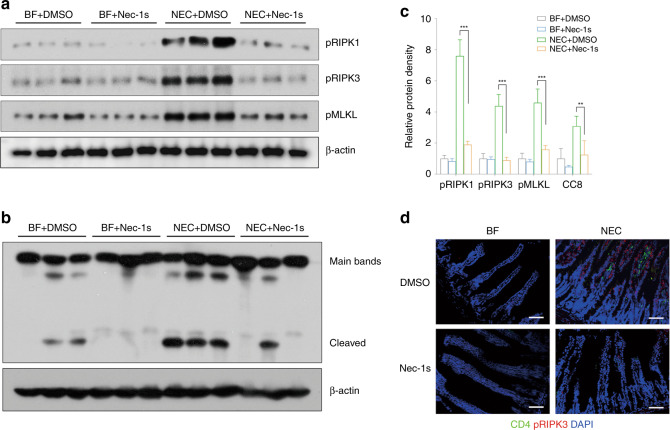
Necrostatin-1s inhibit the expression of necroptotic proteins. **a** Western blotting of necroptosis-related proteins. **b** Western blotting of phosphorylated caspase-8 (cleaved bands). **c** Relative protein density of the above-mentioned proteins. *n* = 6 in each group. **d** Double immunofluorescent staining of CD4 and pRIPK3 in each group. Magnification = ×200, bar = 50 μm. pRIPK1 phosphorylated RIPK1, pRIPK3 phosphorylated RIPK3, pMLKL phosphorylated MLKL, BF breastfeeding under a physiological status, NEC neonatal mice that underwent formula feeding and cold/asphyxia stress, DMSO 10% DMSO in PBS, the dissolvent of necrostatin-1s, nec-1s necrostatin-1s. NEC+DMSO vs. NEC+Nec-1s, **p < 0.01 and ***p < 0.001.

**Fig. 4:**
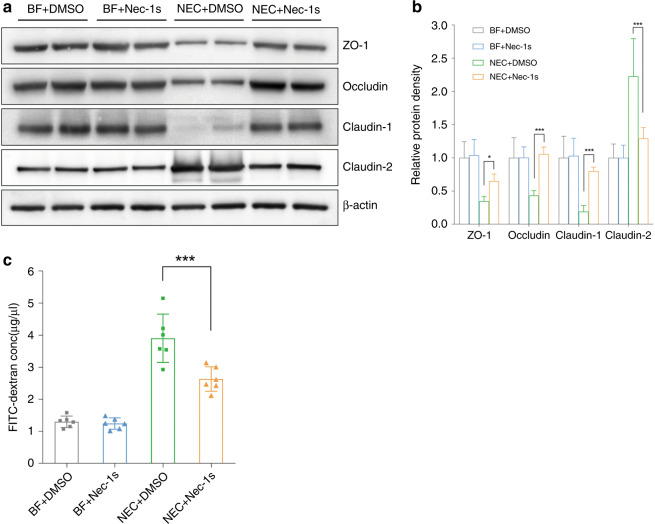
Necrostatin-1s preserved the intestinal barrier function. **a** Western blotting and **b** relative protein density of tight junction proteins. **c** Serum FITC-dextran concentration in each group. *n* = 6 in each group. BF breastfeeding under a physiological status, NEC neonatal mice that underwent formula feeding and cold/asphyxia stress, DMSO 10% DMSO in PBS, the dissolvent of necrostatin-1s, nec-1s necrostatin-1s. NEC+DMSO vs. NEC+Nec-1s, *p < 0.05 and ***p < 0.001.

Since necroptosis might be associated with NEC, we investigated whether necroptosis inhibitors could protect against NEC in animals. Nec-1s significantly increased the survival rates of neonatal mice after NEC induction (*p* < 0.05) (Fig. 2a). Local tissue destruction, which was prominent in NEC mice, was very much alleviated by nec-1s, and the Nadler score decreased significantly (*p* < 0.01) (Fig. 2b, c). NEC challenge increased the mRNA expression of inflammatory cytokines, particularly IL-1β, IL-17, tumor necrosis factor-α (TNF-α), and IFN-γ. This increase was largely inhibited by nec-1s (Fig. 2d). Western blotting showed a dramatic increase in the expression of necroptotic proteins after NEC induction, while this increase was significantly downregulated by nec-1s (*p* < 0.01). (Fig. 3a–c). Decreased necroptosis was accompanied by decreased infiltration of CD4^+^ T cells, indicating fewer inflammatory changes (Fig. 3d). Typical tight junction proteins, such as occludin, ZO-1, and claudin-1, were decreased after NEC induction, while the pore-forming protein claudin 2 was increased. These effects were also ameliorated by nec-1s (Fig. 4a, b). Opening of the barrier in NEC led to the leakage of bowel contents into the circulation, thereby increasing the detectable concentration of FITC-dextran. Nec-1s reduced the amount of leakage and the circulatory FITC-dextran concentration (*p* < 0.001) (Fig. 4c).

### Nec-1s reduced intestinal cell death by reducing necroptosis but increased apoptosis

**Fig. 5:**
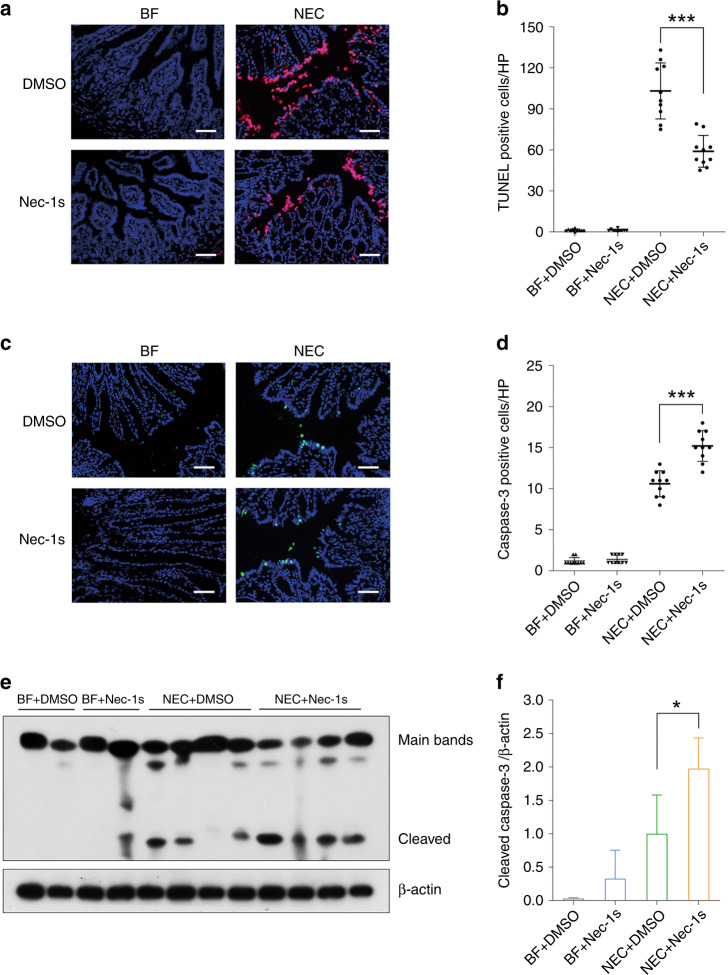
Necrostatin-1s reduced total intestinal cell death but slightly increase apoptosis. **a** Typical TUNEL staining of the proximal colon in each group. Magnification = ×200, bar = 50 μm. **b** Numbers of TUNEL-positive cells per field, *n* = 10 in either group. **c** Typical immunofluorescent staining of cleaved caspase-3 of the proximal colon in each group. Magnification = ×200, bar = 50 μm. **d** Numbers of cleaved caspase-3-positive cells per field, *n* = 10 in either group. **e** Western blotting and **f** relative protein density of cleaved caspase-3. *n* = 4 in either group. BF breastfeeding under a physiological status, NEC neonatal mice that underwent formula feeding and cold/asphyxia stress, DMSO 10% DMSO in PBS, the dissolvent of necrostatin-1s, nec-1s necrostatin-1s, CC3 cleaved caspase-3. NEC+DMSO vs. NEC+Nec-1s, *p < 0.05 and ***p < 0.001.

In the murine model, nec-1s generally reduced the total amount of intestinal cell death caused by NEC, as shown by TUNEL staining (*p* < 0.001) (Fig. 5a, b). However, local expression of cleaved caspase-3, a representative apoptosis mediator, increased after nec-1s administration (*p* < 0.001) (Fig. 5c, d). This effect also occurred in the epithelium. Western blotting confirmed the increase in the cleaved form of caspase-3 after NEC induction and a further increase following nec-1s treatment (*p* < 0.05) (Fig. 5e, f). This finding may indicate a shift from necroptosis to apoptosis.

### TLR4 induced necroptosis through the TRIF and TNFR1 pathways

**Fig. 6:**
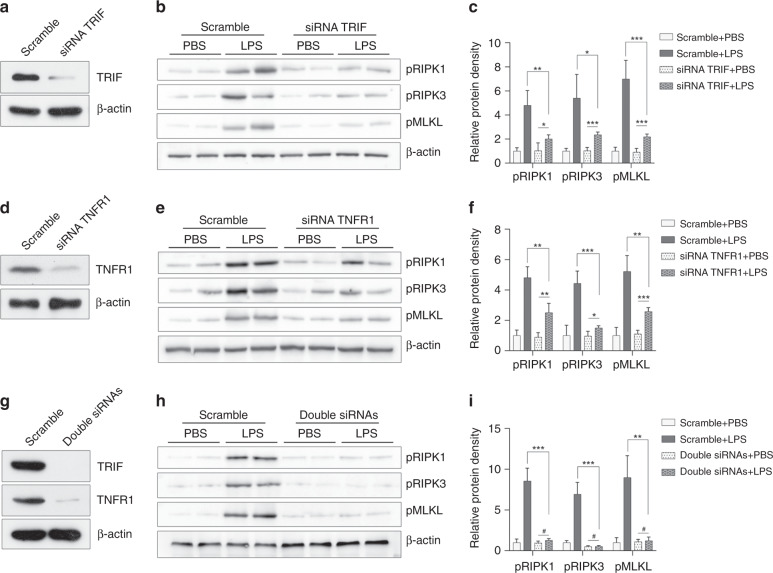
TLR4-mediated necroptosis through the TRIF and TNFR1 pathways. **a** The effect of TRIF siRNA transfection, as confirmed by Western blot analysis. **b** Western blotting and **c** relative protein density of necroptotic proteins after LPS challenge in the scramble and the TRIF silenced HCT-116 cells. **d** The effect of TNFR1 siRNA transfection, as confirmed by Western blot analysis. **e** Western blotting and **f** relative protein density of necroptotic proteins after LPS challenge in the scramble and the TNFR1-silenced HCT-116 cells. **g** Simultaneous TRIF and TNFR1 siRNA transfections resulted in near-complete depletion of both TRIF and TNFR1 expressions, as confirmed by Western blot analysis. **h** Western blotting and **i** relative protein density of necroptotic proteins after LPS challenge in the scramble and the TRIF/TNFR1-silenced HCT-116 cells. pRIPK1 phosphorylated RIPK1, pRIPK3 phosphorylated RIPK3, pMLKL phosphorylated MLKL. ^#^*p* > 0.05, *p < 0.05, **p < 0.01, and ***p < 0.001. *n* = 4 in each group.

There are two major mechanisms that could explain the necroptotic effect of TLR4: the TRIF pathway and the TNFR1 pathway. Therefore, we blocked TRIF and TNFR1 individually and in combination in cells to examine their effects on LPS-induced necroptosis, which is one of the pathological processes in NEC. Blocking TRIF or TNFR1 in HCT-116 cells alleviated LPS-induced necroptosis (Fig. 6a–f). When both pathways were blocked simultaneously, the expression of necroptotic proteins was almost undetectable (Fig. 6g–i). This finding demonstrated that both TRIF and TNFR1 were involved in necroptosis.

### TLR4-knockout ameliorated NEC by inhibiting necroptosis

**Fig. 7:**
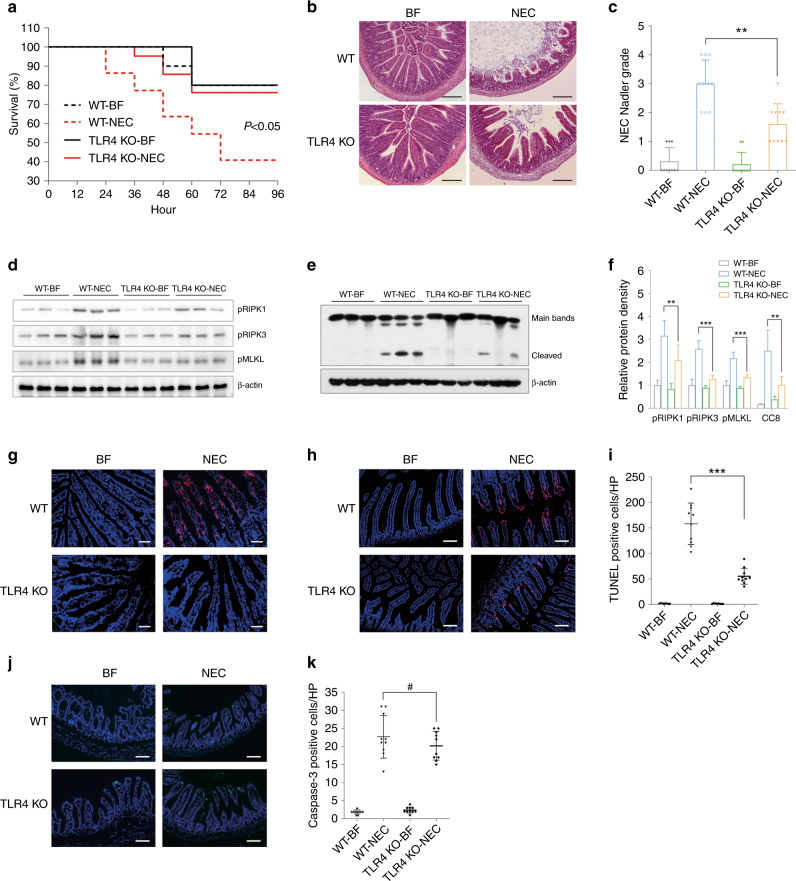
TLR4 knockout protected against NEC partly by inhibiting necroptosis. **a** The survival rate of neonatal mice in each group. *n* = 10 in either of the BF group, *n* = 22 in the WT + NEC group, and *n* = 21 in the TLR4-KO + NEC group. **b** Typical hematoxylin and eosin staining of the distal ileum in each group, magnification = ×100, bar = 100 μm. **c** The Nadler’s grade, *n* = 10 in each group. **d** Western blotting of necroptosis-related proteins. **e** Western blotting of phosphorylated caspase-8 (cleaved bands). **f** Relative protein density of the above-mentioned proteins. *n* = 6 in each group. **g** Typical immunofluorescent staining of phosphorylated RIPK3 in each group. Magnification = ×200, bar = 50 μm. **h** Typical TUNEL staining of the distal ileum in each group. Magnification = ×200, bar = 50 μm. **i** Numbers of TUNEL-positive cells per field, *n* = 10 in either group. **j** Typical immunofluorescent staining of cleaved caspase-3 of the distal ileum in each group. Magnification = ×200, bar = 50 μm. **k** Numbers of cleaved caspase-3 positive cells per field, *n* = 10 in either group. WT wild-type mice, TLR4 KO mice with global TLR4 knockout, BF breastfeeding under a physiological status, NEC neonatal mice that underwent formula feeding and cold/asphyxia stress, CC3 cleaved caspase-3. WT + NEC vs TLR4 KO + NEC, ^#^*p* > 0.05, **p < 0.01, and ***p < 0.001.

Since the incidence of NEC was mediated by TLR4, we induced NEC in TLR4-knockout mice to determine the mechanism. While more than half of the wild-type pups died of NEC, TLR4-knockout pups had a survival rate of ~80% (*p* < 0.05) (Fig. 7a). Intestinal structure destruction, which is a prominent feature of NEC, was very mild in TLR4-knockout pups (*p* < 0.01) (Fig. 7b, c). The sharp increase in pRIPK1, pRIPK3, and pMLKL expression, which was prominent in wild-type pups after NEC induction, was minor in TLR4-knockout pups. Moreover, the level of caspase-8 was decreased (Fig. 7d–f). The increase in pRIPK3 in the intestinal epithelium was also suppressed (Fig. 7g). TUNEL staining did show decreased intestinal epithelial cell death, but caspase-3-mediated apoptosis exhibited was hardly changed, indicating that necroptosis largely contributed to the decrease in intestinal epithelial cell death (Fig. 7h–k).

## Discussion

NEC occurs when the immature intestine fails to adapt well to the outside environment. Incomplete microvasculature development or immature regulation of intestinal vascular tone reduces O_2_ delivery to intestinal epithelial cells. The inadequate intestinal barrier facilitates the colonization and penetration of bacteria. The immature immune system cannot eliminate microbes sufficiently, and the gut or the entire body can initiate an irreversible inflammatory cascade. When triggered by enteral feeding, TLR4, platelet-activating factor (PAF), nitric oxide, and proinflammatory cytokines all work together to induce the drastic pathological process of NEC.^1,2,12,13^

Apoptosis has long been regarded as one mechanism of enterocyte death in NEC.^13^ Excessive apoptosis may exacerbate breakdown of the intestinal barrier, allowing more microbes to enter the submucosal zone and causing a worsened inflammatory response. This response is characterized by the production of TNF-α, IFN-γ, and IL-18, induces apoptosis, and destroys the barrier.^12,14^ However, as an immunologically silent type of cell death, apoptosis itself seems inadequate to explain the rapid, dramatic inflammatory response in NEC. Rather, necroptosis, a proinflammatory mechanism of cell death, exhibits many similarities with NEC. Necroptotic cells quickly release cellular damage-associated molecular patterns and cause a dramatic inflammatory response.^15^ This response may result in epithelial cell death, the loss of Paneth cells, enteritis, and severe erosive colitis, most of which are also features of NEC.^16^

Necroptosis refers to RIPK3-dependent regulated necrosis, which normally occurs when the function of caspase-8 is inhibited. RIPK3 has an RIP homotypic interaction motif (RHIM) domain with which it can bind with RIPK1, DAI, TRIF, and itself.^9^ Typically, necroptosis involves the formation of RIPK1/3 ligands and the subsequent activation of MLKL.^9^ In our study, we observed increases in RIPK1, RIPK3, and MLKL in both NEC patients and model animals. There was significant epithelial sloughing, villus destruction, and even perforation involving the entire thickness of the epithelium. Significant increases in IL-6, IL-17, and TNF-α confirmed the role of T-helper type 1 (Th1)- and Th17-related inflammation in NEC, with the former involved in intracellular pathogens and the latter in extracellular bacterial infections.^17^ The activation of Th1-mediated inflammation influences the severity of NEC,^18^ while the accumulation of CD4^+^ Th17 lymphocytes contributes directly to the development of NEC.^19^ Moreover, our study confirmed that an imbalance in regulatory (Tregs) and effector T cells was a key pathophysiological change in NEC, as indicated by reduced IL-10 production.^20^

To confirm the role of necroptosis, we used the RIPK inhibitor nec-1s to determine whether inhibiting necroptosis could lead to the alleviation of NEC. Nec-1s is a derivative of nec-1 that functions as an allosteric inhibitor of RIPK1 in a T-loop-dependent manner.^21^ While nec-1 has been reported to function through mechanisms other than stabilizing RIPK1, nec-1s targets RIPK1 exclusively with the same effects as nec-1.^22^ In addition, nec-1s does not have the same sensitizing effect as nec-1 when used at a low dose.^22^ Nec-1s inhibits necroptosis by blocking the binding of RIPK1 and RIPK3,^23^ thereby reducing necroptosis and protecting against intestinal injury in NEC model animals. The survival rate almost doubled with less CD4 cell infiltration, showing that overall inflammation was alleviated. The secretion of IL-17 was significantly reduced, while that of IL-10 was slightly increased. This result indicated that the balance between Th17 cells and Tregs had partially recovered. Barrier function was also restored, as there were more tight junction proteins and less pore-forming claudin 2 expression. This result was further confirmed by reduced leakage of bowel contents into the circulation. These findings, together with a previous report,^24^ confirmed that necroptosis was an important mechanism in NEC. Targeting RIPK1 could significantly alleviate pathological changes and might be a promising prevention or treatment for NEC.

Although total intestinal epithelial death was reduced by nec-1s, apoptosis was somehow increased. This finding reflected a shifting balance between necroptosis and apoptosis, which are both present in NEC. RIPK1 is protective against caspase-8-dependent apoptosis, and so RIPK1 inhibition by nec-1s inevitably leads to the activation of apoptosis.^25^ Inhibition of RIPK1 could sensitize intestinal epithelial cells to TNF-α; therefore, although TNF-α secretion was generally reduced, the local effect of TNF-α was exaggerated, contributing to the increase in apoptosis.^25^ However, according to our findings, the pro-inflammatory effect of necroptosis was much stronger than that of apoptosis, and so the beneficial effect of necroptosis inhibition could overwhelm the destructive effect of slight apoptosis activation.

The increase in apoptosis may also be caused by the increase in TLR4 expression.^26^ TLR4 is indispensable in gut development.^1^ Without TLR4, the gut has an abnormal abundance of goblet cells.^27^ Normally, the expression of TLR4 decreases soon after birth, but in NEC patients, TLR4 is increased or at least remains at a high level.^7^ The activation of TLR4 might stimulate the glycogen synthase kinase-3β, nuclear factor-κB (NF-κB), and signal transducer and activator of transcription 3 signaling pathways.^12,28^ TLR4 also functions by promoting the activity of Th17 cells and suppressing the activity of Tregs, thereby causing a dramatic local inflammatory response.^19^ We, as well as others, observed decreased apoptosis in TLR4-knockout mice.^29^ Meanwhile, TLR4 activation inhibited mucosal repair by reducing enterocyte migration and the proliferation of Lgr5-expressing stem cells.^27^

TLR4 also plays a role in the activation of necroptosis. First, increased TLR4 binding to TRIF activates TLR4-mediated necroptosis. On the other hand, the activation of inflammatory pathways, such as the NF-κB pathway, by TLR4 results in increased production of TNF-α, which binds with TNFR1 to activate RIPK1-mediated necroptosis.^9^ This study confirmed the increase in TNF-α after NEC induction. The anti-necroptotic effect of nec-1s might also be partly attributed to the suppression of TLR4.^30,31^ Although TLR4 activation may suppress caspase-8-mediated TNF-associated necroptosis, these two pathways may crosstalk and promote each other to form a vicious loop.^32^

**Fig. 8:**
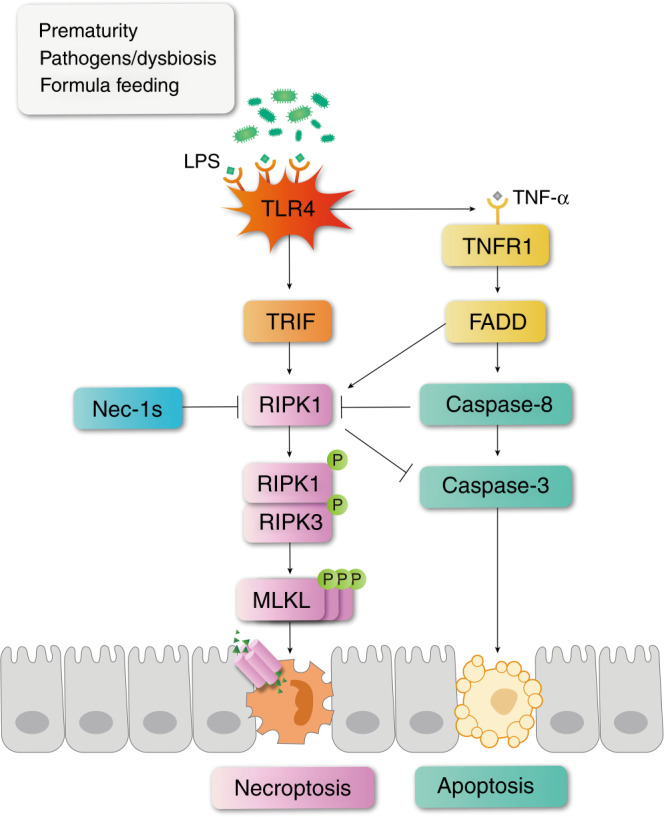
Schematic illustrations of how necroptosis and apoptosis were regulated in NEC. In NEC patients or cells challenged by LPS, TLR4 is activated. On the one hand, TLR4 binds to TRIF to activate necroptosis. On the other hand, TLR4 activation induces the production of inflammatory cytokines including TNF-α, which further binds with TNFR1 and leads to apoptosis and necroptosis simultaneously. When RIPK1 is inhibited by necrostatin-1s, or when TLR4 is removed, necroptosis becomes suppressed. The production of TNF-α is also reduced so that caspase-8 is decreased, leading to a decreasing trend of apoptosis. Meanwhile, the suppression of RIPK1 might relieve its inhibitory effect on caspase-3, leading to an increased trend of apoptosis. These two effects may counteract each other, but in general necrostatin-1 and TLR4 knockout showed an alleviating effect on NEC mice.

TLR4 may execute its biological effects by activating TRIF and myeloid differentiation factor 88 (MyD88). By binding to TRIF, TLR4 may directly induce necroptosis, as TRIF has an RHIM domain that can bind directly with RIPK1.^9^ By binding to MyD88, TLR4 may further activate the NF-κB pathway, leading to increased production of TNF-α, which subsequently activates TNFR1-induced necroptosis. It has been reported that the role of TRIF is more dominant than that of MyD88.^33^ However, according to our findings, both TRIF and TNFR1 contributed to LPS-induced necroptosis. This indicated that TNF-α was still an important stimulator of necroptosis in NEC. However, according to our findings, both TRIF and TNFR1 contributed to LPS-induced necroptosis. This result indicated that TNF-α was still an important stimulator of necroptosis in NEC (Fig. 8).

Removing or inhibiting TLR4 has been reported to be closely associated with the amelioration of necroptosis and the relief of inflammation.^34,35^ This effect has been shown to be protective against LPS-induced intestinal inflammation, which is a model with a mechanism similar to that of NEC.^36,37^ When TLR4 was removed, necroptosis could not be activated to the same degree due to reduced TRIF-mediated necroptosis. The production of cytokines was simultaneously reduced by the reduction in NF-κB pathway activation, which inhibited TNFR-mediated necroptosis. This finding explained the suppression of necroptosis in TLR4-knockout mice after NEC induction. TLR4 knockout suppressed both apoptosis and necroptosis, but the suppression of necroptosis seemed more prominent than that of apoptosis.

However, there might be other mechanisms through which necroptosis is induced or regulated in NEC. One example is PAF, which can simultaneously upregulate TLR4 to induce necroptosis and the NF-κB pathway to suppress necroptosis.^6,38^ Another example is TNF-ɑ, which plays an important but complicated role in the shifting balance between apoptosis and necroptosis.^6^ Clinical investigations have also shown that breast milk protects against NEC partly by inhibiting necroptosis.^24^ Most of the factors involved in NEC are multifunctional and may play dual roles in the development of NEC; thus, further examination of the interactions among several cell death mechanisms in NEC would be interesting.

## Conclusion

Our study confirmed a pivotal role of necroptosis in the pathological process of NEC, which accounts at least in part for dramatically devastating intestinal injuries. By inhibiting RIPK1, a key mediator of necroptosis, injuries could be alleviated, and the survival rate could be elevated. TLR4 may stimulate necroptosis in NEC. Inhibiting intestinal cell necroptosis might be a useful strategy in the treatment of NEC.

## Supplementary information

Supplementary Table 1, 2
