# Supplementary material for: Toll-like receptor 4-mediated necroptosis in the development of necrotizing enterocolitis
Source: Pediatr Res. 2021 Mar 17;91(1):73–82. doi: 10.1038/s41390-021-01457-y (PMC8770135; doi:10.1038/s41390-021-01457-y)
Supplement: Supplementary file 1 — Supplementary Table 1, 2 [file 41390_2021_1457_MOESM1_ESM.pdf]

Supplementary Table 1 Demographic characteristics of the study population

	NEC (n=8)	CIA (n=8)	P value
Gestational age (week), Mean±SD	28.68±1.16	28.75±1.20	0.904
Birth weight (g), Mean±SD	1093.75±146.83	1105.63±124.32	0.864
Days after birth	19.75±4.98	8.63±4.03	0.0002

NEC: necrotizing enterocolitis; CIA: congenital intestinal atresia.

Supplementary Table 2 Primer sequences used for real-time PCR.

Primer name	Forward (5' - 3')	Reverse (3' - 5')
mouse IL1β	CCAAAAGATGAAGGGCTGCT	ACAGAGGATGGGCTCTTCTT
mouse IL6	CCTCTGGTCTTCTGGAGTACC	ACTCCTTCTGTGACTCCAGC
mouse IL10	ATAACTGCACCCACTTCCCA	GGGCATCACTTCTACCAGGT
mouse IL17	TCTCCACCGCAATGAAGACC	CACACCCACCAGCATCTTCT
mouse TNFα	ATGAGCACAGAAAGCATGA	AGTAGACAGAAGAGCGTGGT
mouse IFNγ	TTCTTCAGCAACAGCAAGGC	TCAGCAGCGACTCCTTTTCC
mouse MCP1	GCTCAGCCAGATGCAGTTAA	TCTTGAGCTTGGTGACAAAAACT
mouse p65	GGAGGGAGTTTGGCTCTTTC	CAGTCAGCCATCAGGTGCTA
mouse B2M	CGGCCTGTATGCTATCCAGA	GGGTGAATTCAGTGTGAGCC
